# Therapeutic Effect of Cilostazol Ophthalmic Nanodispersions on Retinal Dysfunction in Streptozotocin-Induced Diabetic Rats

**DOI:** 10.3390/ijms18091971

**Published:** 2017-09-14

**Authors:** Noriaki Nagai, Saori Deguchi, Hiroko Otake, Noriko Hiramatsu, Naoki Yamamoto

**Affiliations:** 1 Faculty of Pharmacy, Kindai University, 3-4-1 Kowakae, Higashi-Osaka, Osaka 577-8502, Japan; 1111610121m@kindai.ac.jp (S.D.); hotake@phar.kindai.ac.jp (H.O.); 2 Laboratory of Molecular Biology and Histochemistry Joint Research Support Promotion Facility Center for Research Promotion and Support Fujita Health University, 1-98 Dengakugakubo, Kutsukake, Toyoake, Aichi 470-1192, Japan; norikoh@fujita-hu.ac.jp (N.H.); naokiy@fujita-hu.ac.jp (N.Y.)

**Keywords:** nanoparticle, cilostazol, diabetic retinopathy, electroretinogram, streptozotocin-induced diabetic rat

## Abstract

We previously prepared ophthalmic formulations containing cilostazol (CLZ) nanoparticles by bead mill methods (CLZ_nano_), and found that instillation of CLZ_nano_ into rat eyes supplies CLZ into the retina. In this study, we investigated changes in the electroretinograms (ERG) of streptozotocin-induced diabetic rats (STZ rats), a model of diabetes mellitus. In addition, we demonstrated that dispersions containing CLZ nanoparticles attenuate changes in the ERG of STZ rats. The instillation of CLZ_nano_ had no effect on body weight or plasma glucose and insulin levels. Furthermore, no corneal toxicity was observed in the in vivo study using STZ rats. The a-wave and b-wave levels in addition to oscillatory potentials (OP) amplitude decreased in STZ rats two weeks after the injection of streptozotocin, with the instillation of CLZ_nano_ attenuating these decreases. In addition, the level of vascular endothelial growth factor (VEGF) in the retinas of STZ rats was 9.26-fold higher than in in normal rats, with this increase also prevented by the instillation of CLZ_nano_ Thus, we have found that a-wave and b-wave levels in addition to OP amplitude are decreased in rats following the injection of excessive streptozotocin. Furthermore, the retinal disorders associated with diabetes mellitus are attenuated by the instillation of CLZ_nano_. These findings provide significant information that can be used to design further studies aimed at developing anti-diabetic retinopathy drugs.

## 1. Introduction

Diabetes mellitus is a chronic disease that affects a large proportion of people worldwide. Diabetic retinopathy (DR) is one of the more common complications associated with diabetes mellitus [1,2,3]. DR is characterized by a progressive alteration in retinal microvasculature [1], which leads to capillary closure and areas of non-perfusion. This eventually results in retinal hypoxia. The non-perfused tissues enhance the production of vascular endothelial growth factor (VEGF) from the retina and causes a subsequent loss of vision and pathologic neovascularization [1]. Previous studies indicate that the changes in electroretinograms (ERG) were observed in the patients with diabetes, even when they have no symptoms of retinopathy [4,5,6]. Moreover, recent studies have found that microstructural changes in the intracranial optic nerve can be observed in the eye of early experimental diabetes before substantial morphological alterations [7,8,9]. In the clinical setting, the development of therapies for retinal dysfunction caused by diabetes mellitus is expected.

Cilostazol (CLZ) has antiplatelet, antithrombotic, and vasodilatory properties [10]. The main pharmacological effect of CLZ is to increase the level of intracellular cyclic AMP through the inhibition of phosphodiesterase 3A [10,11]. CLZ appears to have a favorable effect in preventing the progression of carotid atherosclerosis and intracranial arterial stenosis [12,13]. Hotta et al. [14] reported that treatment with CLZ leads to a vasodilatory effect on retinal arterioles and improved blood supply in animal models of diabetes. Therefore, the reduction in the vascular resistance and increased blood supply to the retina by CLZ may be useful for the treatment of DR.

In developing CLZ as a therapy for DR, it is important to design an ophthalmic formulation that can deliver the drug to the posterior segment. Recently, it has been reported that the formulation containing nanoparticles significantly improved the ability of drugs with regard to corneal penetration [15,16,17,18,19,20]. Furthermore, the formulation may lead an alternative strategy for enhancing ocular drug penetration [21,22,23]. We have also prepared the dispersions containing solid nanoparticles, which can provide high-quantity dispersions containing drug nanoparticles by a simple procedure [24,25,26,27,28,29,30]. Moreover, our previous reports show that dispersions containing CLZ nanoparticles prepared by a bead mill method supply CLZ into the retina through instillation, which can suppress retinal vasoconstriction in 1 × 10^−5^ M endothelin (ET-1)-injected rats (15 μL) [31].

In this study, we investigated changes in ERG of streptozotocin-induced diabetic rat (STZ rat), which is a model of diabetes mellitus. In addition, we studied whether dispersions containing CLZ nanoparticles can attenuate the changes in ERG of STZ rats.

## 2. Results

### 2.1. Changes in Retinal Function in STZ Rats

**Figure 1:**
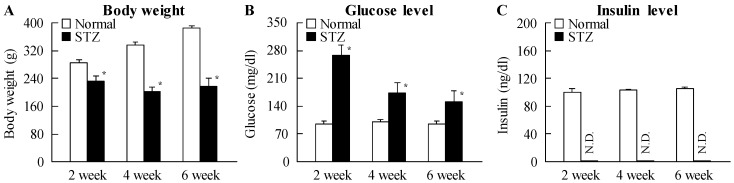
(**A**) Body weight, (**B**) plasma glucose, and (**C**) insulin levels in rats at two, four, and six weeks after the injection of streptozotocin. Samples were collected at 10:00 a.m. Open columns: normal rat; closed columns: streptozotocin-induced diabetic rats (STZ rats) with *n* = 6–8. N.D.: not detectable. * *p* < 0.05, vs. normal rat for each category.

**Figure 2:**
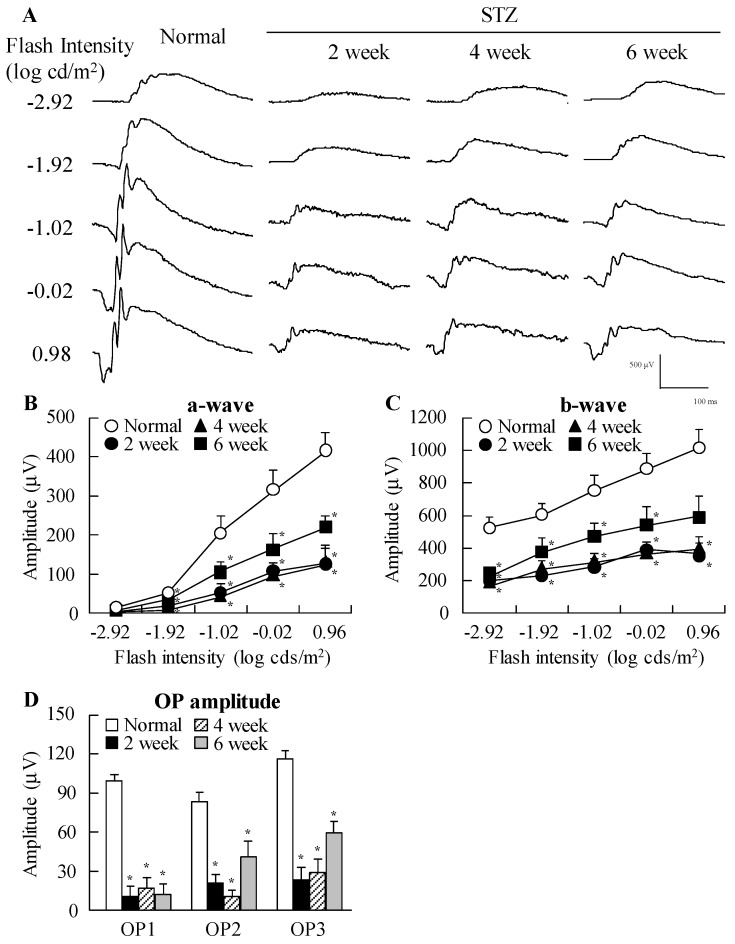
Typical traces of (**A**) electroretinograms (ERG); (**B**) a-wave; (**C**) b-wave; and (**D**) oscillatory potential (OP) amplitude in rat retina at two, four, and six weeks after the injection of streptozotocin. Dark-adapted ERG responses were measured at 2:00 p.m. and stimulus flashes were used from −2.92 log cds/m^2^. *n* = 6–8. * *p* < 0.05, vs. normal rat for each category.

Figure 1 shows the changes in body weight, plasma glucose and insulin levels in STZ rats. The body weight of STZ rats was significantly lower than that of normal rats, while insulin levels were below the level of detection in STZ rat plasma. In addition, glucose levels were enhanced by the injection of streptozotocin, with the plasma glucose levels in STZ rats two weeks after the injection of streptozotocin being 2.8-fold higher than in normal rats. Figure 2 shows the changes in ERG of STZ rats. The levels of a-wave, b-wave, and oscillatory potentials (OPs) amplitude in STZ rats were lower than in normal rats. These retinal disorders were observed at 2–6 weeks after the injection of streptozotocin.

### 2.2. Preventive Effect of CLZ_nano_ Instillation on Retinal Disorders in STZ Rats

**Figure 3:**
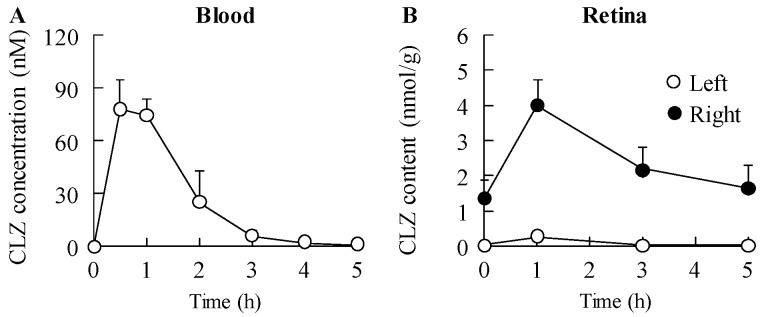
Cilostazol (CLZ) levels in the (**A**) blood and (**B**) retina of rats following the instillation of CLZ_nano_. The streptozotocin was injected on two consecutive days (100 mg/kg × 2, i.p.), after that the instillation of 1% CLZ_nano_ in the right eye of STZ rats were started for two weeks (twice a day, 9:00 a.m. and 7:00 p.m.). The blood and retina were collected at 0–5 h after the last-instillation of CLZ_nano_ (9:00 a.m.–2:00 p.m.); *n* = 7; * *p* < 0.05, vs. vehicle for each category.

**Figure 4:**
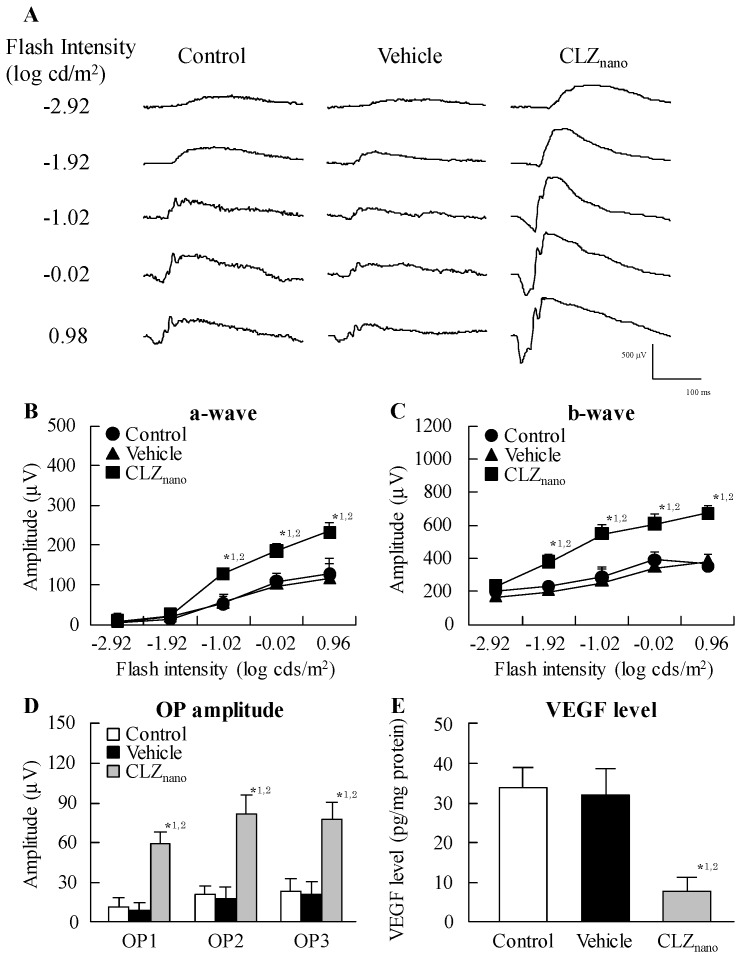
Typical traces of (**A**) electroretinogram (ERG); (**B**) a-wave; (**C**) b-wave; and (**D**) OP amplitude and (**E**) VEGF level in STZ rats after the instillation of CLZ_nano_. The streptozotocin was injected on two consecutive days (100 mg/kg × 2, i.p.), after that the instillation of 1% CLZ_nano_ in the right eye of STZ rats were started for two weeks (twice a day, 9:00 a.m. and 7:00 p.m.). Dark-adapted ERG responses and vascular endothelial growth factor (VEGF) were measured at 5 h after the instillation (2:00 p.m.), and the stimulus flashes in ERG were used from −2.92 log cds/m^2^. Control: non-instilled STZ rat; Vehicle: vehicle-instilled STZ rats; CLZ_nano_ = CLZ_nano_-instilled STZ rats. *n* = 5–7; *^1^ *p* < 0.05, vs. control for each category; *^2^ *p* < 0.05, vs. vehicle for each category.

**Figure 5:**
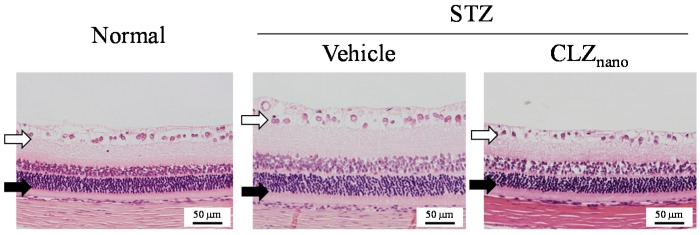
Hematoxylin and eosin (H.E.) staining of retina in normal and STZ rats. The streptozotocin was injected on two consecutive days (100 mg/kg × 2, i.p.), after that the instillation of 1% CLZ_nano_ in the right eye of STZ rats were started for two weeks (twice a day, 9:00 a.m. and 7:00 p.m.). Bars indicate 50 μm. Open arrows: retinal ganglion cells; Normal: normal rat; Vehicle: vehicle-instilled STZ rats; CLZ_nano_: CLZ_nano_-instilled STZ rats; and closed arrows: outer granule layer.

Table 1 shows the changes in body weight in addition to plasma glucose and insulin levels in STZ rats instilled with or without CLZ_nano_. The instillation of CLZ_nano_ had no effect on body weight or on plasma glucose and insulin levels. Figure 3 shows the changes in CLZ levels in the blood and retina of STZ rats after the instillation of CLZ_nano_. A low concentration of CLZ was found in the blood of rats after CLZ_nano_ instillation, while CLZ was also detected in the left eye of instilled rats. On the other hand, the CLZ content in the right eye of rats instilled with CLZ_nano_ was significantly higher than in the left eye. Figure 4A–D show the effect of CLZ_nano_ instillation on ERG of STZ rats. The instillation of CLZ_nano_ attenuated the decrease in the a-wave and b-wave levels, as well as in OP amplitude. Figure 4E shows the changes in VEGF in the retina of STZ rats instilled with CLZ_nano_. The VEGF levels were enhanced by the injection of streptozotocin, with the VEGF levels in STZ rats being 9.26-fold that in normal rats (3.66 ± 0.48, means ± S.E., *n* = 6). The instillation of CLZ_nano_ also prevented the increase in VEGF levels in STZ rats. Figure 5 shows histopathological alterations in the retina of STZ rats detected using a hematoxylin and eosin (H.E.) staining method. The distance between cells was increased in the inner plexiform layer, as well as the outer- and inner-nuclear layer (neural layer) in retinas of STZ rats, while the distance in neural layer was normalized by the instillation of CLZ_nano_ (Figure 5). The distance between cells in the neural layer in STZ rats instilled with CLZ_nano_ was similar to that in normal rats (normal rat 74.1 ± 5.67 µm, STZ rat 116.2 ± 9.90 µm, CLZ_nano_-instilled STZ rat 81.8 ± 7.57 µm, *n* = 5).

### 2.3. Corneal Stimulation by CLZ_nano_ Instillation

**Figure 6:**
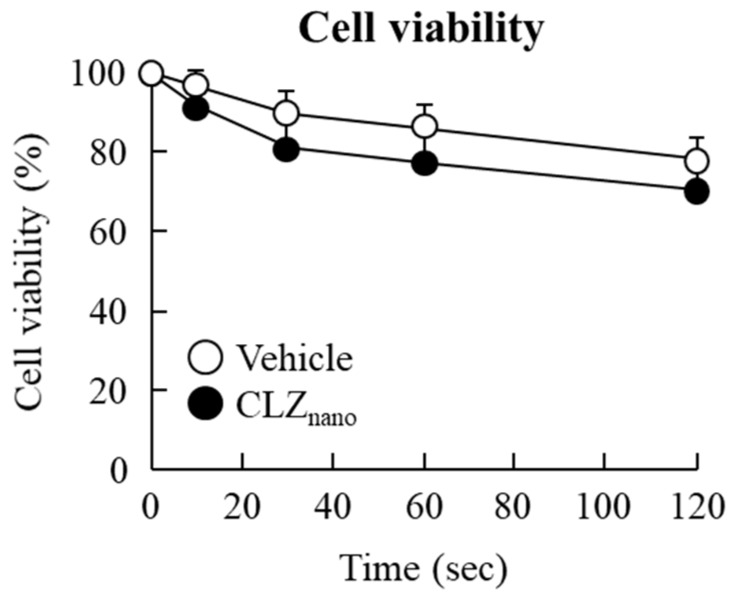
Corneal toxicity of CLZ_nano_ in a human corneal epithelial cell line (HCE-T cells). HCE-T cells in 96-well microplates were treated with vehicle or CLZ_nano_ for 0–120 s and the cell viability was calculated using Tetra-Color One. Vehicle: vehicle-treated HCE-T cells; CLZ_nano_: CLZ_nano_-treated HCE-T cells, *n* = 9.

Figure 6 shows the effect of CLZ_nano_ instillation on the viability of HCE-T cells. The viability of non-treated human corneal epithelial cell line (HCE cells, the cells’ viability without anything added, including both CLZ and vehicle) did not differ for 0–120 s, and there were no significant differences in viability between vehicle- and CLZ_nano_-treated cells. In addition, the corneal stimulation was also evaluated using an STZ rat instilled with CLZ_nano_ for six weeks. Neither the vehicle nor CLZ_nano_ induced corneal epithelium damage (in vivo study).

## 3. Discussion

We previously designed novel ophthalmic formulations containing CLZ nanoparticles (CLZ_nano_), and found that CLZ_nano_ instillation can deliver CLZ into the retina [31]. In this study, we demonstrate the therapeutic effects of CLZ_nano_ on retinal disorders caused by diabetes mellitus in STZ rats.

In both patient and animal models of diabetes, there is a loss of retinal neurons early in the disease progression [32], with this neuronal dysfunction being reflected in alterations in the ERG [33,34]. Moreover, patients with diabetes show altered visually-evoked potentials or ERG even when there is no observed retinopathy [4,5,6]. First, we investigated the changes in retinal function in rats after the injection of streptozotocin by measuring ERG. We show that the a-wave and b-wave levels in addition to the OP amplitude are decreased in rats two weeks after the injection of streptozotocin (Figure 2). There are some previous reports related to measuring the ERG of STZ rats. Li et al. [35] reported that the a-wave and b-wave amplitudes in STZ rats decreased two weeks after the injection of 60 mg/kg streptozotocin. On the other hand, Kohzaki et al. [36] showed that a-wave and b-wave responses were not significantly reduced while OPs were significantly reduced at eight weeks after the injection of 50 mg/kg streptozotocin. Thus, the onset of retinal dysfunction in STZ rats differs depending on the amount of streptozotocin injected and the data suggest that the difference is caused by the severity of diabetes mellitus via the injection of streptozotocin. In this study, plasma insulin was undetectable, while the plasma glucose levels in rats fasted for 12 h was high (STZ rat at two weeks after streptozotocin, 267.6 mg/dL). These results show that the severity of diabetes mellitus in our STZ rats was higher than in previous reports [35,36]. Early changes in ERG may determine the severity of diabetes mellitus via a high-dose injection of streptozotocin (100 mg/kg × 2). It is known that a-waves reflect the function of photoreceptors, b-waves reflect bipolar cell and Müller cell function and OPs are dependent on the hemodynamics in the central retinal artery. Taken together, our findings suggest that the retinas in STZ rats are injured and the retinal dysfunction arose at about two weeks after the injection of streptozotocin.

In treating the posterior segments, it is important to improve the effectiveness of ocular drugs by enhancing their bioavailability [37]. We previously designed a novel ophthalmic formulation called CLZ_nano_ and reported that the state of the CLZ_nano_ does not affect the antimicrobial activity of benzalkonium chloride against *Escherichia coli*. In addition, the instillation of the CLZ_nano_ can deliver CLZ in a therapeutic range into the retina [31] where it suppresses retinal vasoconstriction in 1 × 10^−5^ M ET-1-injected rats (15 μL) [31]. The CLZ_nano_ may lead to their new usage as therapies in the ophthalmologic field. Therefore, we demonstrated the therapeutic effect of CLZ_nano_ instillation on retinal dysfunction in STZ rats. The instillation of CLZ_nano_ had no effect on body weight or on plasma glucose or insulin levels (Table 1). Furthermore, no corneal toxicity was observed in the in vitro and in vivo studies using HCE-T cells or STZ rats (Figure 6). On the other hand, the CLZ content in the retina of the right eye (with instillation) was significantly higher than that of the left eye (without instillation) in rats instilled with CLZ_nano_ (Figure 3). These results support our previous study and it was suggested the *C*_max_ in 1% CLZ_nano_ instillation did not show any systemic effects, such as changes in blood pressure and flow in the carotid artery [38]. Following this, we measured the changes in ERG for STZ rats instilled with CLZ_nano_. The instillation of CLZ_nano_ attenuated the decrease in the levels of the a-wave and b-wave, as well as the OP amplitude in STZ rats (Figure 4). This shows that the instillation of CLZ_nano_ may be useful as a therapy for retinal dysfunction via hyperglycemia.

The measurement of factors associated with the onset of DR in STZ rats, instilled with or without CLZ_nano_, is important with regard to certifying the therapeutic effect. The Müller cells of the retina are activated and upregulate proangiogenic and vascular permeability factors, such as VEGF [39,40], at the onset of DR via retinal hypoxia. These VEGFs contribute to the development of clinical symptoms of retinopathy. In addition, many recent studies have identified the central role of VEGF as a main focus for developing treatments for the vascular lesions observed in DR. Blocking the action of VEGF is a main focus for developing a treatment for this debilitating disease. Therefore, we investigated changes in VEGF levels in the retinas of STZ rats instilled with or without CLZ_nano_. We found that VEGF levels in the retinas of rats are enhanced by the injection of streptozotocin, with the instillation of CLZ_nano_ found to suppress these enhanced VEGF levels in STZ rats (Figure 4E). In addition, we previously reported that retinal hypoxia increases the distance between cells in the inner plexiform layer, the outer- and inner- nuclear layers (neural layer) in the retinas of STZ rats. The enhanced retinal thickening caused by the injection of excessive STZ may lead to retinal dysfunction, resulting in a decrease in ERG [41]. The enhanced retinal thickening was also reversed by the instillation of CLZ_nano_ (Figure 5). These results show that CLZ_nano_ may prevent retinal hypoxia through vasodilatory effects, resulting in a decrease in VEGF production and reversal of the changes in ERG and retinal thickening.

Further studies are needed to determine the mechanism for the ERG and histopathological alterations of rats injected with high-dose streptozotocin. In addition, it is important to clarify the precise mechanism for the preventive effect of CLZ_nano_ on retinal dysfunction. Therefore, we are now investigating the effect of CLZ_nano_ on retinal blood flow in STZ rats using laser Doppler velocimetry and will demonstrate the changes in cyclic adenosine 3′,5′-monophosphate (cAMP) levels in retina after the instillation of CLZ_nano_. On the other hand, the enhanced retinal thickening was not observed in a lower-dose STZ model or a genetic model of diabetic retinopathy, and thus reversal effects of CLZ_nano_ may not necessarily work on diabetic complications. Therefore, it is also important to investigate the effect of CLZ_nano_ on retinal disorders in the genetic model of diabetic retinopathy in future study.

## 4. Materials and Methods

### 4.1. Reagents and Animals

Original CLZ (powder type) and methylcellulose (MC) were kindly donated by Otsuka Pharmaceutical Co., Ltd. (Tokyo, Japan) and Shin-Etsu Chemical Co., Ltd. (Tokyo, Japan), respectively. All other chemicals used were purchased and of the highest purity commercially available. Male Wistar rats (normal rat) were obtained from Kiwa Laboratory Animals Co., Ltd. (Wakayama, Japan). Diabetes mellitus was induced in 6-week old Wistar rats by injecting them with streptozotocin on two consecutive days (100 mg/kg × 2, i.p., STZ rat) and housing them for 0–6 weeks under standard conditions (7:00 a.m.–7:00 p.m. (fluorescent light), 25 °C). All procedures were performed in accordance with the Association for Research in Vision and Ophthalmology resolution on the use of animals in research and the Kindai University Faculty of Pharmacy Committee Guidelines for the Care and Use of Laboratory Animals (identification code: KAPS-25-003, From 1 May 2013).

### 4.2. Preparation of Ophthalmic CLZ Nanodispersions

**Figure 7:**
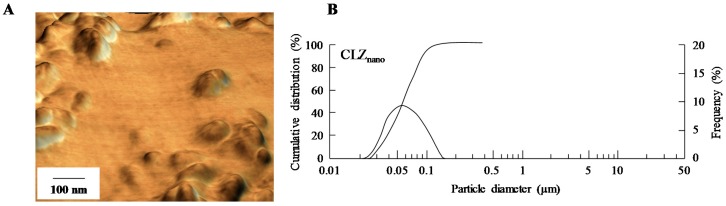
(**A**) Image, in addition to (**B**) the cumulative size distribution and frequency of 1% CLZ_nano_. The image of CLZ_nano_ was obtained on an SPM-9700 and particle size was determined using a SALD-7100 nanoparticle size analyzer (refractive index 1.60–0.10 imaginary unit). Dashed line: the cumulative size distribution; solid line: the cumulative size frequency and mean particle size of CLZ_nano_ is 59 nm.

CLZ solid nanodispersions were prepared using zirconia balls, Pulverisette 7 (a planetary ball mill, Fritsch Japan Co., Ltd., Tokyo, Japan) and Bead Smash 12 (a bead mill, Wakenyaku Co. Ltd., Kyoto, Japan) according to our previous study [31]. CLZ powder, 2-hydroxypropyl-β-cyclodextrin (HPβCD, Nihon Shokuhin Kako Co., Ltd., Tokyo, Japan), benzalkonium chloride (BAC, Kanto Chemical Co., Inc., Tokyo, Japan), mannitol (d-mannitol, Wako Pure Chemical Industries, Ltd., Osaka, Japan) and MC were used to prepare the CLZ nanoparticles. The compositions of the dispersions containing CLZ solid nanoparticles (CLZ_nano_) were as follows: 1% CLZ, 0.001% BAC, 0.1% mannitol, 1% MC, and 5% HPβCD (a pH of 6.5). The solubility of CLZ in saline containing 0.001% BAC, 0.1% mannitol, 1% MC and 5% HPβCD was 0.037% (the solubility of CLZ in saline is 0.0005%). The vehicle in this study used a solution containing these various additives (0.001% BAC, 0.1% mannitol, 1% MC and 5% HPβCD), while the concentration of CLZ used in this study was detected according to our previous study [31]. In the course of preparation, the solvent containing additives was filtered through a Minisart CE (pore size of 0.20 µm, Costar, Cambridge, MA, USA) under aseptic conditions. Images and particle sizes were obtained using an SPM-9700 scanning probe microscope (Shimadzu Corp., Kyoto, Japan) and a SALD-7100 nanoparticle size analyzer (Shimadzu Corp., Kyoto, Japan; refractive index 1.60–0.10 imaginary unit), respectively (Figure 7).

### 4.3. Measurement of CLZ by HPLC

CLZ levels in the samples were determined according to our previous report using a Shimadzu LC-20AT system [31]. Benzophenone was selected as the internal standard, while an Inertsil^®^ ODS-3 column (GL Science Co., Inc., Tokyo, Japan) was used. The mobile phase consisted of acetonitrile/methanol/water (35/15/50, *v*/*v*/*v*) at a flow rate of 0.25 mL/min, the column temperature was 35 °C and the wavelength for detection was 254 nm. The peak of CLZ was detected at 3.7 min (retention time).

### 4.4. Measurement of Plasma Glucose and Insulin

Glucose and insulin (parameters for diabetes mellitus) were measured for normal and STZ rats according to our previous methods [42]. Blood was taken without anesthesia from the tail vein of each rat fasted for 12 h (10:00 a.m.). The plasma glucose and insulin levels were measured by an Accutrend GCT (Roche Diagnostics, Mannheim, Germany) and an ELISA Insulin Kit (Morinaga Institute of Biological Science Inc., Kanagawa, Japan), respectively.

### 4.5. Measurement of CLZ Content in Blood and Retina

Thirty microliters of 1% CLZ_nano_ was instilled into the right eye of the STZ rats twice a day (9:00 a.m. and 7:00 p.m.), before the blood and retina were collected at 0–5 h after the morning instillation (9:00 a.m.–2:00 p.m.). The samples were homogenized in methanol on ice and centrifuged at 10,000 rpm for 15 min at 4 °C. CLZ in the supernatant was analyzed by the HPLC method described above.

### 4.6. Measurement of VEGF

Retinas were collected (2:00 p.m.) and homogenized in 50 mM of Tris buffer on ice, before being centrifuged at 15,000 rpm for 20 min at 4 °C. The supernatants were used for the measurement of VEGF using a rat VEGF immunoassay Quantikine ELISA kit according to the manufacturer’s instructions (R and D Systems, Inc., Minneapolis, MN, USA). VEGF levels are expressed as pg/mg protein. Protein levels in the samples used to determine the VEGF levels were assessed using a Bio-Rad Protein Assay Kit (Bio-Rad Laboratories, Hercules, CA, USA).

### 4.7. Measurement of ERG

ERG readings were recorded by PuREC (Mayo, Aichi, Japan) zero, two, four, and six weeks after the injection of streptozotocin. The rats were maintained in a completely dark room for 24 h, after which they were anesthetized with isoflurane. The pupils were dilated with 0.5% tropicamide and 0.5% phenylephrine (Santen, Osaka, Japan). Flash ERG was recorded in the right eyes of the dark-adapted rats by placing a golden-ring electrode (Mayo, Aichi, Japan) in contact with the cornea and a reference electrode (Mayo, Aichi, Japan) through the tongue. A neutral electrode (Mayo, Aichi, Japan) was inserted subcutaneously near the tail. All procedures were performed under dim red light. The amplitude of the a-wave was measured from the baseline to the maximum a-wave peak, while the b-wave was measured from the maximum a-wave peak to the maximum b-wave peak. The a-wave shows the function of the photoreceptors, while the b-wave reflects bipolar cell and Müller cell function. To analyze the oscillatory potentials (OPs), the OP amplitudes were measured in the time between the a- and b-wave peaks. The relevant factors were OP number (OP1, OP2, and OP3) and flash intensity (0.98 (log cds/m^2^)). OPs were isolated by the band pass filter and OP amplitudes were measured using ERG with all frequencies (0.3–500 Hz). The OPs were dependent on the hemodynamics in the central retinal artery.

### 4.8. Morphology of Rat Retina

Whole rat eyes were fixed in SUPER FIX™ rapid fixative solution (Kurabo Industries, Osaka, Japan) and 3-μm paraffin sections were prepared from the fixed whole rat eyeballs in the usual manner [43]. The rat retinal tissue was observed in detail by hematoxylin and eosin (H.E.) staining. A microscope (Power BX-51, Olympus, Tokyo, Japan) was used for observation. The distance from the retinal ganglion cell to the outer granule layer (neural layer: inner plexiform layer, outer- and inner-nuclear (granule) layer) was calculated using Image J (NIH, MD, USA). The photographed area has a position of about 4–5 o’clock or 7–8 o’clock when the center of the cornea is at the 12 o’clock position in the sagittal section of the eyeball at approximately the middle part of the optic nerve and the peripheral part of the retinal nerve.

### 4.9. Measurement of In Vitro Corneal Epithelial Stimulation by CLZ_nano_

The experiment was performed according to our previous study using the immortalized human corneal epithelial cell line, HCE-T [27]. HCE-T cells (1 × 10^4^ cells) were seeded in 96-well microplates (IWAKI, Chiba, Japan) and one day after seeding, the cell cultures were stimulated by 1% CLZ_nano_ for 0–120 s. The time was determined according to a previous report as the components of eye drops are excreted though the nasolacrimal duct into the mouth at approximately 120 s after instillation [44]. Following stimulation, a culture medium containing TetraColor One (SEIKAGAKU Co., Tokyo, Japan) was added and incubated for 1 h. After that, the absorbance (Abs) at 490 nm was measured and cell viability was calculated according to the manufacturer’s instructions, as represented by Equation (1):

Cell viability (%) = Abs_treatment for each group_/Abs_non-treatment for each group_ × 100 (1)

The wash-off and medium change was done in the non-treatment groups, and the Abs in non-treatment groups were similar.

### 4.10. Measurement of In Vivo Corneal Toxicity by CLZ_nano_

Thirty microliters of 1% CLZ_nano_ was instilled into the right eyes of STZ rats twice a day (9:00 a.m. and 7:00 p.m.) for six weeks (repetitive instillation). The eyes were kept open for about 1 min after instillation to prevent the 1% CLZ_nano_ from being washed out. The wound area (corneal epithelial damage) was stained with 1% fluorescein (Alcon Japan, Tokyo, Japan) and measured using a TRC-50X fundus camera (Topcon, Tokyo, Japan) equipped with a digital camera (the instillation of fluorescein can stain the corneal epithelium damage). The image was obtained 5 h after the instillation (2:00 p.m.).

### 4.11. Statistical Analysis

Unpaired Student’s *t*-test, Aspin-Welch’s *t*-test or Dunnett’s multiple comparison was used, with *p* < 0.05 considered significant. All data are expressed as means ± S.E.

## 5. Conclusions

We found that a-wave and b-wave levels, in addition to OP amplitude, were decreased in rats following the injection of excessive streptozotocin, while retinal disorders associated with diabetes mellitus were attenuated by the instillation of CLZ_nano_. These findings provide significant information that can be used to design further studies aimed at developing anti-diabetic retinopathy drugs.

## Figures and Tables

**Table 1:** Body weight and plasma glucose and insulin levels in STZ rats after the instillation of ophthalmic formulations containing cilostazol nanoparticles (CLZ_nano_).

Treatment	Body Weight (g)	Glucose (mg/dL)	Insulin (ng/dL)
Vehicle	301.1 ± 8.68	273.2 ± 30.7	N.D.
CLZ_nano_	299.5 ± 9.10	269.7 ± 29.8	N.D.

One percent CLZ_nano_ was instilled into the right eyes of STZ rats twice a day (9:00 a.m. and 7:00 p.m.) for two weeks. The samples were collected 1 h after the instillation (10:00 a.m.), *n* = 7, and N.D.: not detectable.

## Abbreviations

Abs	Absorbance
BAC	Benzalkonium Chloride
CLZ	Cilostazol
DR	Diabetic Retinopathy
ERG	Electroretinogram
ET	Endotheline
HCE-T	Human Corneal Epithelial Cell Line
H.E.	Hematoxylin and Eosin
HPβCD	2-Hydroxypropyl-β-Cyclodextrin
OPs	Oscillatory Potentials
MC	Methylcellulose
STZ rat	Streptozotocin-Induced Diabetic Rat
VEGF	Vascular Endothelial Growth Factor
